# The Efference Copy Signal as a Key Mechanism for Consciousness

**DOI:** 10.3389/fnsys.2021.765646

**Published:** 2021-11-26

**Authors:** Giorgio Vallortigara

**Affiliations:** Centre for Mind/Brain Sciences, University of Trento, Rovereto, Italy

**Keywords:** efference copy, corollary discharge, consciousness, sensation/perception, sensory reafference

## Abstract

Animals need to distinguish sensory input caused by their own movement from sensory input which is due to stimuli in the outside world. This can be done by an efference copy mechanism, a carbon copy of the movement-command that is routed to sensory structures. Here I tried to link the mechanism of the efference copy with the idea of the philosopher Thomas Reid that the senses would have a double province, to make us feel, and to make us perceive, and that, as argued by psychologist Nicholas Humphrey, the former would identify with the signals from bodily sense organs with an internalized evaluative response, i.e., with phenomenal consciousness. I discussed a possible departure from the classical implementation of the efference copy mechanism that can effectively provide the senses with such a double province, and possibly allow us some progress in understanding the nature of consciousness.

## Introduction

*La música, los estados de felicidad, la mitología, las caras trabajadas por el tiempo, ciertos crepúsculos y ciertos lugares, quieren decirnos algo, o algo dijeron que no hubiéramos debido perder, o están por decir algo; esta inminencia de una revelación, que no se produce, es, quizá, el hecho estético*.

*Jorge Luis Borges*

Since its description by von Holst and Mittelstaedt (1950) and Sperry (1950), the idea that the efference copy signal may play a crucial role in consciousness has been put forward by several authors (see for an historical account Grüsser, 1995; Fukutomi and Carlson, 2020).

The concept of an efference copy arose in the framework of the problem of space constancy, i.e., the fact that the visual world appears stable despite shifts of overall visual input with eye movements. Anticipations of the idea can be found in several authors, such as Bell (1823), Purkinje (1825), von Helmholtz (1866), von Helmholtz and Southall (1962), and von Uexküll (1920), (see Koenderink, 2015) but the breakthrough came from seminal experiments by Erich von Holst and Roger Sperry.

von Holst and Mittelstaedt (1950) inverted the head of the blowfly *Eristalis*, holding it with a piece of wax. The fly appeared to circle either clockwise or counterclockwise at random. Given that in the darkness the fly’s movement looked pretty normal, they argued for the existence of a mechanism that compared the output of the locomotor system with the retinal flow field. von Holst and Mittelstaedt (1950) hypothesized an «*Efferenzkopie*» that would be compared and subtracted from the retinal signal to stabilize locomotion. Tilting the head converted the ordinary negative feedback of the efference copy into a positive feedback—a motor command in one direction would feed back a signal to correct in the same direction, thus giving rise to further deviation in the same direction and continuous circling as a result. Sperry (1950) made similar observations in an independent way, studying fish with surgically inverted eyes, and named the signal «corollary discharge». Although distinctions have been proposed in the literature for use of the two terms (Li et al., 2020), in this article I will use efference copy and corollary discharge interchangeably.

The efference copy signal may enable organisms that move to discount sensory stimulation that arises from their own actions, thereby allowing them to distinguish between the sensory stimulation caused by external stimuli and that caused by their own movements.

Irwin Feinberg (1978) first suggested that failures of the efference copy mechanisms may underlie some of the symptoms of psychosis. This was then developed by Frith (1987) and Shergill et al. (2005). Specifically, Feinberg (1978) argued that dysfunction of efference copy mechanisms that normally allow us to recognize and disregard stimulation resulting from our own actions would characterize schizophrenia, giving rise to the subtle but pervasive sensory/perceptual aberrations observed in these patients. Disturbances of the efference copy mechanisms may contribute to symptoms such as hallucinations and delusions: a failure to recognize one’s voice or inner speech as self-generated might produce the subjective experience of an externally generated sound, thus giving rise of auditory hallucination of hearing voices; or a failure to predict the sensory consequences of one’s actions may result in the subjective experience of being under the control of external forces.

The mechanisms of the efference copy was then slowly absorbed into the general framework of predictive coding with the idea that the brain needs to infer the causes of a given sensory input, which can be achieved through combining new sensory data with pre-existing knowledge of the world or priors (Ford and Mathalon, 2019). However, several authors have stressed a specific role of efference copy mechanisms on the origins of consciousness (Merker, 2005; Godfrey-Smith, 2016, 2020; Vallortigara, 2021a).

In a recent article, Jékely et al. (2021) argued for a role of *Reafference*, i.e., any effect on an organism’s sensory mechanisms that is due to the organism’s own actions, to the evolution of the *body-self*, a form of organization that would enable an animal to sense and act as a single unit. The authors noted that reafference in general does not necessarily involve a nervous system: self-initiated activities tend to have predictable consequences, and reafference would simply represent feedbacks concerning such predictions. An example they discussed comes from sponges, in which sensory cilia keep track of the flow produced within the body and can signal when this flow ceases (Ludeman et al., 2014). They argued for a further evolution of the mechanism of reafference when, in animals with nervous systems, sensory and effector devices made available a more sophisticated engine that compensates for predicted sensory changes by registering the particular action underway at a time.

What is unclear in all these accounts is how reafference or efference copy can give rise to consciousness, i.e., to the feelings that accompany and characterize (at times) our responding to sensory stimulation. I believe some progress on this issue can be made if we try to link the idea of the efference copy with the old-fashioned distinction between sensation and perception of some philosophical traditions.

## Sensation and Perception

In the *Essays on the Intellectual Powers of Man* Thomas Reid (1941) says that «*When I smell a rose, there is in this operation both sensation and perception. The agreeable odour I feel, considered by itself without relation to any external object, is merely a sensation… Its very essence consists in being felt; and when it is not felt it is not. There is no difference between the sensation and the feeling of it—they are one and the same thing… in sensation there is no object distinct from the act of the mind by which it is felt-and this holds true with regard to all sensations (pp. 150–151)»*.

Of course, the terms sensation/perception are associated with a long tradition of debates and different meanings in philosophy (see e.g., Reeves and Dresp-Langley, 2017) but here I am considering only the particular conception developed by this author because of its possible links with biological facts. According to Reid «*The external senses have a double province —to make us feel, and to make us perceive. They furnish us with a variety of sensations, some pleasant, others painful, and others indifferent; at the same time they give us a conception and an invincible belief of the existence of external objects… Sensation, taken by itself, implies neither the conception nor belief of any external object. It supposes a sentient being, and a certain manner in which that being is affected; but it supposes no more. Perception implies a conviction and belief of something external—something different both from the mind that perceives, and the act of perception. Things so different in their nature ought to be distinguished»* (Reid, 1895 [1785], II, Ch. 17 and 16).

Consider the classical example by Reid. When we smell a rose there would be two separate but parallel things happening; namely we feel the sweet smell as a conscious experience (sensation) and we detect the external presence of the object rose (perception). Reid (1895) [1785], II, Ch. 17 and 16) argues that we do not notice or attend to our sensations except under rather special circumstances: «*The mind has acquired a confirmed and inveterate habit of inattention to them, for they no sooner appear than quick as lightning the thing signified succeeds, and engrosses all our regard. They have no name in language; and although we are conscious of them when they pass through the mind, yet their passage is so quick and so familiar, that it is absolutely unheeded* (pp. 135)».

Humphrey (1992, 2006, 2011) beautifully conceptualized the distinction between sensation and perception in terms of representing «what is happening to me» (the feeling of the smell of the rose) and «what is happening out there» (the perception of the object rose). He agrees with Reid that for the most part we overlook our sensations because we focused on the objects of perception. There are, however, clinical conditions that made the sensation/perception distinction apparent. This has been worked out by Humphrey himself, starting from his seminal discovery of the blindsight phenomenon while studying recovering of visual function in the blind monkey Helen (Humphrey and Weiskrantz, 1967). Blindsight patients can recognize «what is happening out there» but their perception is not accompanied by any conscious feeling, i.e., they lack sensation or the «what is happening to me» (Humphrey, 1992).

Humphrey also moved further from Reid in arguing that having a sensation is not a passive condition but rather a form of active engagement with the stimulus occurring at the body surface. He wrote «*When, for example, I feel pain in my toe, or taste salt on my tongue, or equally when I have red sensation at my eye, I am in effect reaching out to the site of stimulation with a kind of evaluative response—a response appropriate to the stimulus and the body part affected. Indeed what I experience as my sensation of “what is happening to me” is based not on the incoming information as such but rather on the signals I myself am issuing to make the response happen*» (Humphrey, 2000).

## The Principle of Reafference as The Foundation of The Sensation/Perception Distinction

There are then two questions. First, why should a distinction between sensation and perception be necessary in evolutionary terms? Second, what sort of mechanism can support the distinction between sensation and perception?

As to the first point, the crucial role of active movement has been stressed as lying at the origin of the development of nervous systems (e.g., Llinás, 2001). Active movement also implies the kind of problem that makes necessary the development of an efference copy. As stated by Merker (2005): «*Consider the worm’s initiation of a crawling movement. Such a movement will produce sudden stimulation of numerous cutaneous receptors (…), yet no withdrawal reflex is released to abort the movement. Apparently the worm’s simple nervous system discounts cutaneous stimulation contingent on self-produced movement as a stimulus for withdrawal*».

Thus, one can see the problem of distinguishing «what is happening to me» from «what is happening out there» as a selective pressure that arose specifically with active movement, and the efference copy as the mechanism which has developed through natural selection as a solution of this specific problem.

So far so good but it remains quite a puzzle why sensation (following Reid and Humphrey) should be associated with consciousness (Note that I am referring here to consciousness—which is a word with high polysemy—as simply «experience», i.e., following Block (1995): «*Phenomenal consciousness is experience; the phenomenally conscious aspect of a state is what it is like to be in that state*».). If we take the model of the efference copy we can easily understand why the sensory signal produced by a local stimulation can be annihilated when an efference copy is generated as a result of the active movement of the organism; however, we cannot understand why a sensation would be there in the absence of any active movement, for when an object is impinging on our surface we do feel something (something happening to us).

My proposal is simply to take seriously the hypothesis put forward by Reid and Humphrey and link it with a sort of reversed principle of reafference (see also Hesslow, 2012 for a similar reversed principle, though not linked to sensation and experience). Essentially, the principle of reafference establishes that the organism is able to predict the sensory consequence of its own action, that is, the stimulation that might occur as a result of its own movement. However, one could also consider the situation the other way around: that the body is able to predict the type of motor consequence, that is, of bodily reaction, which should follow from its sensory activity. Indeed, this is exactly what happens, if we assume that the sensation is actually a bodily reaction, a motor action in itself. The double province of the senses might be established by an efference copy of the motoric aspect (the bodily reaction) of the response to the stimulus. Let’s examine this hypothesis in more detail in the next section.

## Consciousness as Imminence of A Revelation

In the traditional view, the efference copy is a solution to the problem of maintaining the stability of the visual world. So, when for instance an organism moves its eyes, the sliding of retinal images would be canceled by the efference copy associated with the motor command sent to the eye muscles.

**Figure 1:**
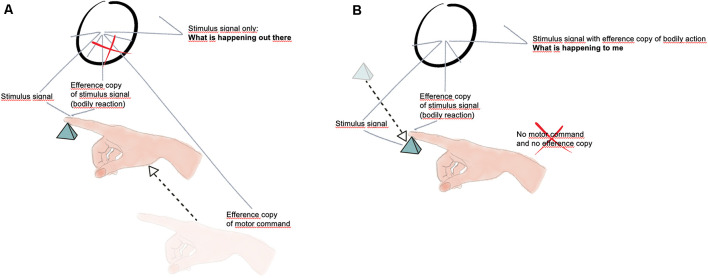
**(A)** The movement of the hand is associated with an efference copy that annihilates the efference copy associated with the local bodily reaction, thus giving rise to the perception of an object out there without sensation (Drawing by Elena Lorenzi). **(B)** The object is moving and hits the finger; in the absence of active hand movement there is no efference copy to annihilate the efference copy associated with the local bodily reaction, thus a sensation (what is happening to me) arises (Drawing by Elena Lorenzi).

Let’s consider a slightly different mechanism, arising from some simple experimental phenomenology as shown in Figures 1A,B. When we move actively an arm to encounter an object, such as the small pyramid in Figure 1A, the active tactile stimulation on the finger is usually associated with the perception of something (an object) out there.

It is quite difficult in these circumstances to focus instead on the feeling of something *on* the finger (which agrees with Reid idea that we do not usually notice or attend to our sensations; and see also more recently Kilteni et al., 2020).

In the reverse condition, however, when the object is moved and hits the finger passively stimulating it, we usually feel something happening to the finger, something happening to us, a sensation (Figure 1B).

It seems to me that this can be conceptualized by arguing that sensory stimulation has indeed a double province, namely that the sensory signal is usually associated with a carbon copy of it (an efference copy) which is escorting the sensory signal thus giving rise, as a bodily action, to a sense of agency, i.e., to the fact that such a sensory signal is produced by the organism itself for it is a motor action, a bodily response. If the touching is the result of an active movement of the arm, then the motor signal associated with this movement would nullify the efference copy (the bodily signal) of the local stimulation. The sensory signal would emerge in this case naked from the comparator, giving rise to a perception (something out there) without any sensation (something happening to me). On the contrary, the impinging stimulation caused by the motion of the object itself that hits the finger would be not associated with any cancellation of the efference copy (bodily signal), thus charging the sensory signal of a sense of authorship, what we describe as feeling or experiencing something (The lack of a sense of authorship is probably a crucial aspect of the behavior of blindsight patients, that need to be convinced «to guess»—such a «motivation/reason for action» could have been another basic outcome of the appearance of the double province of senses.).

Although the model would fit with phenomenal experience for tactile stimulation, it may appear a little paradoxical with distant senses: Do we sometimes really not see (in the sense of sensing, feeling it) when looking at the visual world? Well, yes, certainly we do not sense (feel) anything during saccades, i.e., again when the efference copy associated with the bodily action of visual sensing [of «sentition» as Humphrey (1992) dubbed it] is nullified by the efference copy associated with saccadic movements.

Of course, I am not arguing here that the mechanism (nowadays) is peripheral and local. In the scheme argued for by Humphrey (1994), the body’s senses produced a local response on the body surface in early organisms but then the response becomes targeted on the incoming sensory nerves and finally privatized in an internal brain circuit. However, my point here is that if the local bodily reaction is not associated with a carbon copy of it to be compared with others motor command as it happens in actively moving organisms, no sensation and no feeling (consciousness) would exist. Similarly, I would not expect sensation to occur in sessile organisms (Vallortigara, 2021b).

Borges wrote (see original text *in esergo*) that «*imminence of a revelation, which is not produced, is, perhaps, the aesthetic event*». This can be used as a metaphor for the reafference theory of consciousness described here, i.e., as a sensory signal which is waiting for a bodily action revelation that may or may not occur (Vallortigara, 2020, 2021a). The operating of the comparator (schematized by the circle in Figures 1A,B) that takes into account the different signals likely needs a delay line for the sensory signal of the sort that have been hypothesized in mechanisms such as the Reichardt detector (see Hassenstein and Reichardt, 1956). This time delay could be the foundation of the minimum time duration of the experienced present, an idea dating back to William James (1890) who stressed on the necessity for neural activity to have a suitable duration in order for consciousness to arise from sensory stimulation.

There are advantages in hypothesizing that the comparator would operate on two motoric signals rather than on a sensory and a motoric signal as in the traditional view of the reafference principle (see e.g., for vision Bridgeman, 2010), for we can account better for the phenomenology of our experience and avoid issues that arose with different models of consciousness. Consider for example the ideas put forward by Taylor (1999) who has tried to use the idea of a temporal delay in another way, assuming that the efference copy signal is retained in a temporal memory and that its brief permanence, before its annihilation, would constitute consciousness. In order to do this, Taylor introduced the hypothesis that the corollary discharge is no longer simply derived from the motor signal, but from attention. This corollary discharge of the movement of attention would be retained in a working memory by supplying the properties of experience to the sensory signal before being canceled by it (see Taylor, 2002, 2003).

According to Taylor’s model, consciousness is identified with an efference copy of the attention movement control signal residing briefly in its buffer until the associated attended input activation is also arriving in the buffer. The difficulty, however, is that the attributes of the experience in this framework do not seem to belong to the sensory signal itself, but to the corollary discharge (or to the attentional movement control signal of it). In our example of the hand or the object that moves, the sense of ownership, and of being the agent (the author) of the sensation, would therefore refer to the movement of the finger (or to the attention to the movement of the finger) rather than to the sensation encountered. And in the event that the hand does not move at all but instead is the finger that is passively stimulated by the object due to a displacement and a contact produced by the object itself, there would be no sensation because no attentional movement control signal arises, though sensation is actually happening. Of course, one can argue that besides the efference copy as a potential attentional source, other canonical forms of attention (as heavily investigated in the literature, not necessarily related to motor activity) would be available and thus that the inference from Taylor’s theory to no sensation in the absence of no movement would be probably unfair. Nonetheless, claiming for an efference copy of the movement of attention would be problematic also because evidence suggest that consciousness can be observed without attention, and *vice versa* (Koch and Tsuchiya, 2012). These difficulties dissolve, however, if we evaluate the sensory signal for what it is, or better for what it must have been originally as hypothesized by Humphrey (1992), namely a bodily reaction—a movement in itself—with the possibility of making of a carbon copy of it, in the form of an efference copy.

## Discussion

In general terms, the reafference principle refers to any kind of effect on an organism’s sensory mechanisms that is due to the organism’s own actions. It clearly requires some form of motion of the body but as noted by Jékely et al. (2021) «*even a sessile animal can act with reafferent consequences, as when a filter-feeding animal generates a feeding current by motile cilia*». Yet, it seems to me that only the more advanced form of reafference claimed for by Jékely et al. (2021) can be associated with sensation (as opposed to perception), and thus with consciousness. Single cell organisms such as bacteria can use motility to assess the presence of a chemical gradient. Jékely et al. (2021) describe for example a simple form of deformational reafference with an internal reciprocal influence between the sensory events and the effector. However, it is only with the appearance of specialized sensors and effectors that there would be a specific neural signal to convey reafferent sensing during action. In the example I discussed in Figure 1 involving active touch there is certainly deformational reafference, changes in the shape of the body (at the finger) that lead to sensing. But in order for this sensing to be felt, i.e., to be a sensation, a minimal structure with a sensory neuron, a motor neuron and an interneuron is needed to allow the signal provided by the sensory neuron to be charged (or not to be charged) with the carbon copy (the efference copy) of the motor signal (the deformational bodily reaction) thus providing it with a sense of agency and authorship.

Mechanisms of efference copy have been described at several levels in both vertebrates and invertebrates (Crapse and Sommer, 2008). I would be inclined to consider their presence as a signature of the ability of these organisms to inhabit, as proposed by Reid, a double province of sensory stimulation, that of sensation and that of perception, or in Humphrey’s terminology of «what is happening to me» and «what is happening out there». Of course, all this tells us nothing about the specific contents of the sensations of others organisms. Animals with efference copy mechanisms, I would maintain, should be phenomenally conscious, though the contents of their sensations may be incommensurable to each other, for their origins lay in their species-specific bodily reactions on their different body districts.

Objections can be raised of course to the idea that the double province of the senses might be established by an efference copy of the motoric aspect (the bodily reaction) of the response to the stimulus, and several theoretical aspects certainly need more elaboration. Consider the following examples (see e.g., Owen, 2017 for a review on these topics).

First, mental imagery. There is no stimulus during mental imagery. However, according to the cognitive neuroscience literature of mental imagery, the nervous system would be activated similarly as processing a stimulus. How would mental imagery fit in the distinction of «sensation» and «perception», and how does an efference copy contribute to mental imagery? Second, anesthesia would cause dissociation of action and sensation. Would anesthesia produce an illusion of «sensation» and «perception» that are indistinguishable? Third, an extreme case is the locked-in patients who completely lose movement ability. Would the locked-in patients not smell a rose?

I believe that with respect to these three examples we need to consider the changes that occurred in evolutionary history. At the start sensation was a bodily reaction at the very surface of early organisms (with its efference copy), but then, as stressed by Humphrey (1992, 2000) the local response has become privatized, first by targeting it to incoming sensory nerves and then being entirely located into the brain. Consider again in this regard Feinberg’s (1978) ideas about psychosis: thought processes themselves can be considered as motor actions, as argued by Hughlings Jackson (1958), because, I would say, they are retaining their characteristics of an, albeit privatized, bodily reaction and thus have an efference copy, the lack of which may produce schizophrenic symptoms (the patient is no longer the author of the bodily reaction, i.e., the author of his own thoughts). Thus, imagery, anesthesia and lock-in do not pose a problem for feeling something, assuming that there is an internal motor command that is the internalized version of the original bodily reaction at the organism’s surface.

Several other important issues remain of course unanswered. For example, Reid’s definition of perception does involve some difficulties (see Reeves and Dresp-Langley, 2017). How does one know that an animal believes in the object in front of it? It seems unlikely that fixation of belief is exclusively human. Alex, an African Gray Parrot could tell in a sort of vocal labeling resembling English what he experienced and believed to be present, even including perceptual illusions (Pepperberg, 2002). However, a variety of perceptual illusions have been investigated in non-human animals using traditional motor responses (Vallortigara, 2004, 2006, 2021c; Rosa-Salva et al., 2014), and there seems to be no reason to assume that these motor responses should have a reduced epistemic value with respect to the vocal labeling of Alex (or, for that matter, with respect to human vocal labeling). Clearly, any further discussion should be placed under the light of insight from animal behavior, since the core assumption of this article implies that animals have evolved in strict association with active movement the beginnings of what we call phenomenal experience.

## Data Availability Statement

The original contributions presented in the study are included in the article, further inquiries can be directed to the corresponding author.

## Author Contributions

The author confirms being the sole contributor of this work and has approved it for publication.

## Conflict of Interest

The author declares that the research was conducted in the absence of any commercial or financial relationships that could be construed as a potential conflict of interest.

## Publisher’s Note

All claims expressed in this article are solely those of the authors and do not necessarily represent those of their affiliated organizations, or those of the publisher, the editors and the reviewers. Any product that may be evaluated in this article, or claim that may be made by its manufacturer, is not guaranteed or endorsed by the publisher.
